# Optimizing naloxone distribution to prevent opioid overdose fatalities: results from piloting the Systems Analysis and Improvement Approach within syringe service programs

**DOI:** 10.1186/s12913-023-09289-8

**Published:** 2023-03-22

**Authors:** Sheila V. Patel, Lynn D. Wenger, Alex H. Kral, Kenneth Sherr, Anjuli D. Wagner, Peter J. Davidson, Barrot H. Lambdin

**Affiliations:** 1 Center for Behavioral Health Epidemiology, Implementation, and Evaluation Research, Community Health Research Division, RTI International, 3040 E. Cornwallis Rd, Research Triangle Park, NC 27709 USA; 2 Department of Global Health, University of Washington, Seattle, WA USA; 3 Department of Epidemiology, University of Washington, Seattle, WA USA; 4 Department of Industrial & Systems Engineering, University of Washington, Seattle, WA USA; 5 Department of Medicine, Division Global Public Health, Herbert Wertheim School of Public Health and Human Longevity Science, University of California San Diego, San Diego, CA USA; 6 Department of Epidemiology & Biostatistics, University of California San Francisco, San Francisco, CA USA

**Keywords:** Opioid overdose, Naloxone, Syringe Service Programs, Implementation Strategy, Systems Analysis and Improvement Approach, Interrupted Time Series

## Abstract

**Background:**

Opioid overdose fatalities are preventable with timely administration of naloxone, an opioid antagonist, during an opioid overdose event. Syringe service programs have pioneered naloxone distribution for potential bystanders of opioid overdose. The objective of this study was to pilot test a multi-component implementation strategy—the systems analysis and improvement approach for naloxone (SAIA-Naloxone)—with the goal of improving naloxone distribution by syringe service programs.

**Methods:**

Two syringe service programs participated in a 6-month pilot of SAIA-Naloxone, which included (1) analyzing program data to identify gaps in the naloxone delivery cascade, (2) flow mapping to identify causes of attrition and brainstorm programmatic changes for improvement, and (3) conducting continuous quality improvement to test and assess whether modifications improve the cascade. We conducted an interrupted time series analysis using 52 weeks of data before and 26 weeks of data after initiating SAIA-Naloxone. Poisson regression was used to evaluate the association between SAIA-Naloxone and the weekly number of participants receiving naloxone and number of naloxone doses distributed.

**Results:**

Over the course of the study, 11,107 doses of naloxone were distributed to 6,071 participants. Through SAIA-Naloxone, syringe service programs prioritized testing programmatic modifications to improve data collection procedures, proactively screen and identify naloxone-naïve participants, streamline naloxone refill systems, and allow for secondary naloxone distribution. SAIA-Naloxone was associated with statistically significant increases in the average number of people receiving naloxone per week (37% more SPP participants; 95% CI, 12% to 67%) and average number of naloxone doses distributed per week (105% more naloxone doses; 95% CI, 79% to 136%) beyond the underlying pre-SAIA-Naloxone levels. These initial increases were extended by ongoing positive changes over time (1.6% more SSP participants received naloxone and 0.3% more naloxone doses were distributed in each subsequent week compared to the weekly trend in the pre-SAIA Naloxone period).

**Conclusions:**

SAIA-Naloxone has strong potential for improving naloxone distribution from syringe service programs. These findings are encouraging in the face of the worsening opioid overdose crisis in the United States and support testing SAIA-Naloxone in a large-scale randomized trial within syringe service programs.

Contributions to the literature

- SAIA-Naloxone was an acceptable and feasible implementation strategy to optimize community-based naloxone distribution within two syringe services programs that participated for 6 months.
- SAIA-Naloxone provided opportunities for syringe service program staff to use data to inform decision-making and programmatic modifications that improve access to naloxone among its participants.
- SAIA-Naloxone improved access to naloxone, thereby increasing chances that people who use drugs can reverse overdoses.
- SAIA-Naloxone might be an effective strategy to address disparities in naloxone distribution.

## Background

An estimated 2.2 million people in the United States (US) have an opioid use disorder, and less than 20% of them are currently receiving treatment [1, 2]. In addition, opioid-related overdose mortality has increased over six-fold since 1999 with particularly steep increases in recent years [3–7]. Between 2013 and 2019, the age adjusted rate of deaths involving synthetic opioids other than methadone increased by 1,040% [6]. Alarming increases across demographics indicate that the opioid overdose epidemic continues to surge in the US [8].

Opioid-related overdose deaths are preventable with naloxone, a medication that displaces opioids from the brain receptors to which they attach, reversing the opioid-induced respiratory depression that leads to a fatal overdose [9]. A prescription medication, but not a controlled substance, naloxone is safe, produces no analgesic or euphoric effect, has no potential for abuse, and has been used for more than four decades to reverse opioid overdose [10]. Because the likelihood of permanent injury or death increases with the amount of time a person remains in respiratory depression, it is imperative that naloxone be administered as soon as an overdose is suspected [11]. Increasing access to naloxone in at-risk communities by educating and training potential bystanders—such as people who use drugs, their family members, and their peers—about the symptoms of overdose, how to use naloxone, and the importance of keeping it on their person can ensure naloxone is available and deployed to save lives [12, 13].

Syringe services programs (SSPs), which provide access to and disposal of sterile syringes and injection equipment for people who use drugs, are ideal venues for naloxone distribution as they reach people who are at high risk of observing or experiencing opioid overdose and have staff who are culturally competent in providing services for people who use opioids [14]. While SSPs pioneered naloxone distribution, their distribution of naloxone has not reached sufficient levels to reduce population-level opioid overdose mortality [15–17]. A recent study of people who inject drugs (PWID) found that half (49.4%) had witnessed an overdose and 15.9% had experienced an overdose in the past six months—but only 35.1% currently possessed naloxone [18]. For naloxone to effectively reduce the number of opioid overdose fatalities, a higher proportion of potential bystanders need to be knowledgeable about and consistently possess and use naloxone [12]. Leveraging implementation strategies to strengthen the ability of SSPs to distribute naloxone to their participants is paramount to addressing the nation’s opioid overdose crisis.

As a multi-component implementation strategy, the systems analysis and improvement approach (SAIA) has the potential to improve SSP-based naloxone distribution by centering analysis of service gaps, mapping service flow, and continuous quality improvement. The original SAIA trial focused on the prevention of mother-to-child HIV transmission cascade, where antiretroviral therapy coverage increased threefold and screening for HIV-exposed infants increased 17-fold [19]. Additionally, health workers in the trial described the intervention as easy to use and practical [20]. In the present study, we piloted and assessed an adapted version of SAIA to improve SSPs’ naloxone delivery cascade and increase access to naloxone in communities at high risk of opioid overdose.

## Methods

### Clinical intervention

At the individual-level, naloxone is an effective opioid antagonist that can prevent opioid overdose mortality when administered quickly and appropriately during an opioid overdose event [9, 21]. Efforts to educate and equip potential bystanders of opioid overdose to administer naloxone are highly effective at preventing opioid overdose mortality and have demonstrated cost-effectiveness [12, 22].

The naloxone delivery cascade refers to a series of linked, sequential steps considered necessary for naloxone programs to fully realize the life-saving benefits of the intervention at the population-level. These steps include screening individuals who present for syringe services for naloxone engagement, training potential bystanders who have not been previously engaged on how to reverse an overdose with naloxone, equipping trained bystanders with naloxone to use during observed overdoses, and refilling their supply of naloxone as needed. If followed, these steps could maximize the effectiveness of naloxone by ensuring bystanders have immediate access to naloxone and can administer it when needed.

### Setting

Our study included two SSPs with ongoing naloxone distribution in California. SSPs provide access to and disposal of syringes and injection equipment for PWID and offer a range of other prevention services [14]. They are ideal settings for naloxone distribution as they have staff who are culturally competent in providing services for PWID, and PWID already engage with and trust these organizations to care for their health. In California, SSPs are authorized by the California Department of Public Health. State authorization also allows SSP staff and volunteers to lawfully distribute and possess syringes and naloxone for distribution. California SSPs acquire naloxone from the California Department of Health Care Services. In total, the California Department of Public Health has authorized 61 SSPs in California as of November 2021; all of them distribute naloxone.

Two SSPs with naloxone programs located in large urban centers in California were enrolled. The first SSP (Site 1) was a standalone non-profit organization that began distributing syringes in 1993 and naloxone in 2000. In 2020, site 1 had 8 full time staff, 3 part time staff and 4 volunteers. The organization had an annual budget of $876,869, with funding from their county health department, private foundations, and other community-based organizations. In 2019 and 2020, they served approximately 3,000 participants annually. In addition to providing syringes and naloxone, this SSP provides on-site HIV and HCV counseling and testing; fentanyl test strips; nutritional support; abscess and wound care; individual risk reduction counseling and linkage to substance use treatment.

The second SSP (Site 2) was part of a county health department that began distributing syringes in 1994 and naloxone in 2017. In 2020, site 2 had 3 full time staff and 2 part time staff. The organization had an annual budget of $319,518 with funding from the county and state health departments. SSP services are provided across the county five days a week at five pop-up sites by county employees. Across 2019 and 2020, they served approximately 700 participants annually. In addition to providing syringes and naloxone, this SSP provides on-site STD, HIV, and HCV testing; fentanyl test strips; and referrals to mental health, and substance use treatment.

### Implementation strategy

For this study, we adapted the original SAIA implementation strategy for naloxone delivery within SSPs (SAIA-Naloxone) by directly engaging staff at the two SSPs that were participating in the 6-month pilot study [19, 20, 23, 24]. We held three meetings with staff involved in naloxone distribution and information collection from each of the SSPs (*n* = 6 at Site 1; *n* = 5 at Site 2) to orient them to SAIA and, using principles of focus group discussions, elicit ways in which it should be adapted. Key adaptations for SAIA-Naloxone that resulted from these meetings involved modifying data collection instruments, primarily to reduce data collection burden and align data elements with the Naloxone Cascade Analysis Tool (NCAT) [24]. Briefly, NCAT is an Excel-based simulation model that uses routine data collection to identify gaps in an SSP’s naloxone delivery cascade [24, 25]. NCAT includes the number of SSP participants who presented for syringe services; screened for prior naloxone engagement; of those who were not engaged previously, received training for and a supply of naloxone; and among those who were engaged previously, whether participants still possessed naloxone, had immediate access to naloxone, and/or received a naloxone refill. NCAT guides improvement efforts by promptly identifying which step in this process offers the greatest potential cascade gain to improve completion of the cascade (i.e., improve fidelity to the naloxone delivery cascade) and achieve effective naloxone coverage among SSP participants (i.e., increase service penetration of naloxone) [26].

Like the original SAIA, SAIA-Naloxone included [1] analyzing program data to identify gaps in the naloxone delivery cascade, [2] flow mapping to identify causes of attrition and brainstorm programmatic changes for improvement, and [3] conducting continuous quality improvement to test and assess whether modifications improve the cascade. For example, we first presented baseline data to the SSP staff using NCAT to identify steps in the cascade that could benefit most from intervention (e.g., few participants who were not previously engaged for naloxone receive training). We then led the staff in visually mapping out their service structure, including documenting how different service elements (e.g., registration, supply pick up, naloxone training and refills, and other services) fit together, where staff or volunteers are positioned, and how participants flow through and engage with the different elements. This helps the SSP staff identify where bottlenecks start to develop and where participants start to leave before receiving all appropriate service elements, serving as the basis for understanding drop-offs identified via NCAT (e.g., participants end up leaving after getting screened for naloxone engagement instead of waiting for staff to become available to provide training) and identifying potential solutions to address those drop-offs (e.g., increase staffing for naloxone training). We worked with staff to prioritize solutions that were perceived to be both important in addressing key drop-offs and feasible to adopt, develop a plan for operationalizing prioritized solutions, and evaluate the impact of the adopted solutions using NCAT (e.g., whether fewer participants drop-off between getting screened for naloxone engagement and receiving training after increasing staffing). SAIA-Naloxone is described in detail according to the Proctor guidelines for specifying and reporting implementation strategies in Table 1 [27].

**Table 1:** SAIA-Naloxone implementation strategy

Name it		Systems Analysis and Improvement Approach – Naloxone (SAIA-Naloxone)
Define it		Facilitate development of a quality monitoring system to conduct cyclical small tests of change led by the organizational implementation teams
Specify it	Actor	External facilitator works and facilitates discussion with the organization’s implementation team with regards to the SAIA-Naloxone process
	Actions	1. Identify gaps: a. Present data evaluating the SSP’s naloxone delivery cascade with NCAT b. Facilitate discussions and support the implementation team to identify and develop consensus with regards to the areas of attrition along the cascade that they would like to address 2. Identify causes and opportunities: a. Facilitate discussions with the implementation team to review the SSP’s service structure and draw process maps documenting the flow of participants through the naloxone delivery cascade to understand (i) why there are drop-offs at different points (root causes of participant attrition) and (ii) what it would take to address those issues (opportunities to streamline workflows and address key points of attrition) b. Assist team in developing consensus about programmatic modifications based on their importance and feasibility 3. Conduct continuous quality improvement: a. Support and mentor the implementation team in operationalizing programmatic modifications b. Present follow-up data on the naloxone delivery cascade for the implementation team to assess changes resulting from programmatic modifications c. Repeat above actions after conclusion of the cycle
	Action target	Leverage programmatic data to facilitate continuous quality improvement and foster a learning climate
	Temporality	After training the implementation team on the SAIA process and integrating enhanced instruments to collect program data into workflows to track naloxone delivery cascade indicators
	Dose	Visit the SSP twice during the first month (to identify gaps, causes, opportunities) and once a month in months two to six (to allow enough time for the programmatic modifications to be adopted and for their theoretical impact to show up); each visit lasting approximately 60 min
	Targeted implementation outcomes	1. Improve completion of the cascade (i.e., fidelity to the naloxone delivery cascade) 2. Achieve effective naloxone coverage among SSP participants (i.e., service penetration of naloxone)
	Justification	SAIA-Naloxone combines a broad view of the service system with iterative improvement cycles in a user-friendly way; by leveraging SAIA-Naloxone, SSPs can identify fillable gaps in the naloxone delivery cascade and apply locally generated solutions that have a higher likelihood of leading to measurable and sustained improvements in fidelity to the cascade and penetration of naloxone

### Data collection

#### Primary outcomes

Our primary outcomes included the weekly number of people receiving naloxone and the weekly number of naloxone doses distributed. Improvements in these outcomes signal improvements along the naloxone delivery cascade and reflect improvements in effective naloxone coverage among SSP participants. Further, we focus on these two outcomes as prior research has demonstrated that these metrics significantly predict opioid overdose mortality rates at the community-level [28, 29]. SSPs provided retrospective data for the 52 weeks prior to initiating SAIA-Naloxone. Prospectively, these data were securely transferred to the study team monthly for the 26 weeks after SAIA-Naloxone was initiated.

#### SAIA-Naloxone implementation and process data

We marked each SSP’s SAIA-Naloxone start date as the week following our first meeting with the organization’s implementation team to complete actions 1 through 3 of Table 1. From this point, the SSP was considered actively participating in SAIA-Naloxone. Throughout delivery of the SAIA-Naloxone implementation strategy, the external facilitator prospectively documented which steps of the cascade the SSP implementation team prioritized, the content of programmatic modifications employed by the team, and the change in percent completion of the prioritized cascade steps after implementing the modifications.

### Analysis

To assess the association between SAIA-Naloxone and access to naloxone among communities served by the SSPs, we conducted an interrupted time-series analysis [30]. We utilized population averaged Poisson models where site and week were specified as the panel and time variable, respectively. We constructed a segmented model to assess if SAIA-Naloxone resulted in a change in both level and trend (i.e., slope) in the weekly number of SSP participants receiving naloxone and the weekly number of naloxone doses distributed. Interrupted time-series enables detection of whether SAIA-Naloxone was associated with a significant one-time change and/or shift in weekly trend in the outcomes beyond what is expected given pre-SAIA-Naloxone trends. The model also accounted for potential serial correlation of measures within each of the SSPs over time. The basic equation used for estimating the association between SAIA-Naloxone (I) and each outcome (Y) was:

$$\mathrm{Yst}\:=\:\beta0\:+\:\beta1(\mathrm t)\:+\:\beta2(\mathrm{Is})\:+\:\beta3(\mathrm{Is}\ast\mathrm t)\:+\:\beta4(\mathrm{Xs})\:+\:\varepsilon\!\mathrm{st}.$$

The coefficient on the time indicator (β_1_) provides the pre-SAIA-Naloxone trend for the outcome. For this analysis, this covers the 52-week period prior to the first SAIA-Naloxone meeting the SSP provider. A significant coefficient on the intervention indicator (β_2_) would indicate a one-time change in the outcome beyond the pre-period trend due to SAIA-Naloxone. A significant coefficient on the interaction between the intervention and time (β_3_) would indicate a shift in the outcome trend in the post-period from the pre-period due to initiating SAIA-Naloxone. The models were adjusted for SSP participant volume as a continuous variable and season as a 4-level categorical variable (X). We report 2-sided p-values and considered *p* < 0.05 the cut-off for statistical significance. Statistical analyses were performed using Stata 16.0 (StataCorp, 2021).

## Results

Across the 78-week study period and two SSPs, a total of 20,530 SSP visits occurred with 6,071 participants receiving naloxone and a total of 11,107 naloxone doses distributed. This shifted from a weekly average of 129 SSP participant visits, 17 people receiving naloxone, and 38 naloxone doses distributed prior to SAIA-Naloxone to a weekly average of 127 SSP participant visits, 64 people receiving naloxone and 109 naloxone doses distributed after SAIA-Naloxone.

Twelve staff from across the two SSPs were engaged in the SAIA-Naloxone implementation strategy as part of their SSP implementation team. Six cycles of SAIA-Naloxone were completed as intended across the two SSPs, half of which focused on improving naloxone possession (Table 2). Each modification resulted in at least a 50% improvement in the targeted cascade step. One of the modifications targeting naloxone possession resulted in a 110% improvement for Black, indigenous, and people of color.

**Table 2:** Programmatic modifications and change achieved through SAIA-Naloxone cycles

Naloxone Delivery Cascade Step	Programmatic Modification	Change to Cascade Step
Screening for naloxone	Increased staffing for naloxone screening at exchange sites	70% improvement in number of naloxone screenings conducted
Naloxone training	Developed system to proactively screen participants for prior training experience	51% improvement in number of naloxone trainings conducted
Naloxone refill	Provided naloxone refills with other syringe supplies	78% improvement in number of people receiving naloxone refills
Possess naloxone	Provided reusable bags with pocket for naloxone	60% improvement in number of people possessing naloxone
Possess naloxone	Educated and reminded participants about carrying naloxone	110% improvement in number of people possessing naloxone
Possess naloxone	Allowed secondary distribution of naloxone by participants	116% improvement in number of black, indigenous and people of color possessing naloxone

**Fig. 1:**
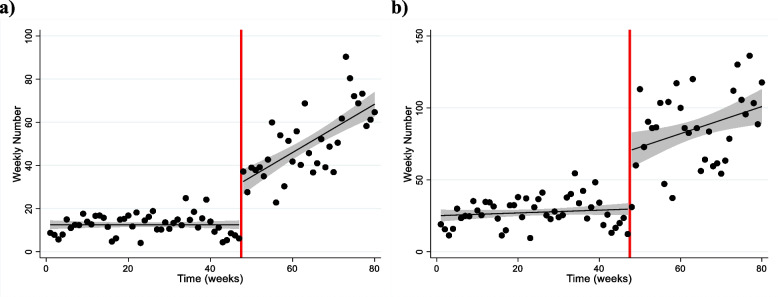
Interrupted time series of the number of (**a**) people receiving naloxone and (**b**) naloxone doses distributed before and after initiating SAIA-Naloxone. Dots represent the pooled (**a**) number of participants receiving naloxone or (**b**) naloxone doses distributed each week. Black lines represent a linear model fit to the data points in the pre- and post-SAIA-Naloxone periods. The introduction of SAIA-Naloxone is represented by the red vertical line

In the adjusted statistical model, SAIA-Naloxone was associated with a significant and substantial change in the weekly number of participants receiving naloxone and number of naloxone doses distributed (Table 3). Prior to introducing SAIA-Naloxone, 1.1% fewer SSP participants received Naloxone each week. Immediately after SAIA-Naloxone was introduced, 37% more SSP participants received naloxone (β = 1.369; 95% CI, 1.122 to 1.669). In each subsequent week, 1.6% more SSP participants received naloxone (β = 1.016; 95% CI, 1.012 to 1.020), compared to the weekly trend in the pre-SAIA Naloxone period. In addition, prior to introducing SAIA-Naloxone, the number of naloxone doses distributed each week remained steady. Immediately after SAIA-Naloxone was introduced, 105% more naloxone doses were distributed (β = 2.055; 95% CI, 1.792 to 2.358). In each subsequent week, 0.3% more naloxone doses were distributed (β = 1.003; 95% CI, 1.001 to 1.005), compared to the weekly trend in the pre-SAIA Naloxone period. Figure 1 depicts the initial changes and changes in trend associated with SAIA-Naloxone.

**Table 3:** Estimates from an interrupted time series segmented regression model

	Pre-SAIA-Naloxone	Post-SAIA-Naloxone		
	Trend (slope)	Change in level	Change in trend	Trend (slope)^a^
	Estimate (95% CI)	Estimate (95% CI)	Estimate (95% CI)	Estimat e (95% CI)
Weekly number of participants receiving naloxone	0.989 (0.986, 0.994)	1.369 (1.122, 1.669)	1.016 (1.012, 1.020)	1.005 (1.002, 1.009)
Weekly number of naloxone doses distributed	1.000 (0.997, 1.002)	2.055 (1.792, 2.358)	1.003 (1.001, 1.005)	1.002 (1.000, 1.005)

*CI* Confidence interval

^a^The post-SAIA-Naloxone trend is the linear combination of the pre-SAIA-Naloxone trend and the post-SAIA-Naloxone change in trend

## Discussion

The results of this pilot demonstrate that the SAIA-Naloxone implementation strategy can improve access to naloxone among SSP participants. The SSPs engaged in 6 cycles of SAIA-Naloxone (3 per SSP) during the implementation period, suggesting that SAIA-Naloxone was acceptable and feasible as an implementation strategy. In addition, the introduction of SAIA-Naloxone at SSPs was associated with statistically significant increases in the number of participants receiving naloxone each week and the number of naloxone doses distributed each week. Further, SAIA-Naloxone was associated with significant increases in the trend of these outcomes, with more participants receiving naloxone and more doses distributed each week. Based on these findings, SAIA-Naloxone has strong potential to optimize the naloxone delivery cascade for SSP participants and prevent opioid-involved overdose deaths.

Prior studies have demonstrated that Black and Latinx PWID were 25% and 47% less likely, respectively, to receive naloxone compared to white PWID [18]. As part of this pilot, a programmatic modification employed by one of the SSPs showed an improvement in naloxone possession among Black, indigenous, and people of color. In addition to assessing for general improvements in naloxone distribution, future trials of SAIA-Naloxone should assess whether it can improve equitable access to naloxone.

Beyond these outcomes, it is possible that SAIA-Naloxone improved aspects of the internal environment of the SSPs. For example, external facilitators engaged the full implementation team which could have shifted power dynamics and allowed front-line staff the opportunity to express their ideas about what is contributing to drop-offs in the naloxone delivery cascade and potential ways to improve them. This could provide an opportunity for innovative, prioritized approaches to emerge from any of the staff members, not just SSP leadership, potentially contributing to a strengthened culture within the organization. Further, leveraging data to engage in continuous quality improvement through multiple cycles of SAIA-Naloxone could foster a learning mindset among SSP staff and might improve the implementation climate for naloxone. Future research should evaluate the impact of SAIA-Naloxone on key implementation determinants like organizational culture and implementation climate and the extent to which they operate as mechanisms through which SAIA-Naloxone improves access to naloxone.

It is important to consider the findings of this study in light of the emerging body of research regarding factors internal and external to SSPs that are related to larger scale naloxone distribution. Results from two national surveys of SSPs have identified that higher levels of community support, more sustained implementations, larger annual budgets, more days of service, and being a non-governmental entity are associated with higher levels of naloxone distribution [17, 31]. While SAIA-Naloxone may strengthen the organizational culture and implementation climate within an SSP, it does not address several of the structural-related characteristics that are related to larger scale distribution. Longer term studies of SAIA should explore whether effects plateau based on structural factors of the SSP, and if encountered, which supplemental approaches can improve these structural factors [32]. Even with these constraints, SAIA-Naloxone was able to improve naloxone distribution within these SSPs.

While the findings from this pilot study are promising, there are some limitations to consider. In piloting SAIA-Naloxone, we did not randomly select SSPs, randomly assign SSPs to receive SAIA-Naloxone, nor were we able to use a design with a robust control. Still, the use of an interrupted time series approach allowed us to assess changes within SSPs and provided an estimate of the outcomes above and beyond what would have been expected in the absence of SAIA-Naloxone [32]. This design limits concern for confounding due to between site differences, but the possibility of confounding due to co-occurring interventions or events remains a possibility. Further, through this pilot, SAIA-Naloxone was deemed feasible by external facilitators and SSP implementation teams, and the effect estimates are strong enough to warrant a randomized trial. Lastly, pre-period outcome data were reported retrospectively by the participating SSPs; however, SSPs involved in the study maintained electronic records of these data for routine reporting and could easily transfer them to the study team. The limitations of this pilot study are being addressed through a randomized controlled trial currently underway, which requires SSPs to report baseline data for a lead-in period before randomizing them to receive SAIA-Naloxone or implementation as usual and collects data beyond the intervention period to assess the extent to which impacts of SAIA-Naloxone are sustained.

## Conclusions

SAIA-Naloxone is a feasible implementation strategy that leverages programmatic data and principles of continuous quality improvement to increase access to naloxone among SSP participants who are likely bystanders of opioid-related overdoses. Ensuring this population is prepared and equipped to reverse overdoses and prevent fatalities is essential to combatting the worsening opioid overdose crisis in the US.

## Data Availability

The dataset created and analyzed during the current study are available from the corresponding author on reasonable request.

## Abbreviations

HCV: Hepatitis C virus
HIV: Human immunodeficiency virus
NCAT: Naloxone Cascade Analysis Tool
PWID: People who inject drugs
SAIA: Systems Analysis and Improvement Approach
SSP: Syringe services program
STD: Sexually transmitted disease
US: United States
